# Recombination-Mediated Host Adaptation by Avian *Staphylococcus aureus*

**DOI:** 10.1093/gbe/evx037

**Published:** 2017-04-01

**Authors:** Susan Murray, Ben Pascoe, Guillaume Méric, Leonardos Mageiros, Koji Yahara, Matthew D. Hitchings, Yasmin Friedmann, Thomas S. Wilkinson, Fraser J. Gormley, Dietrich Mack, James E. Bray, Sarah Lamble, Rory Bowden, Keith A. Jolley, Martin C.J. Maiden, Sarah Wendlandt, Stefan Schwarz, Jukka Corander, J. Ross Fitzgerald, Samuel K. Sheppard

**Affiliations:** 1 Swansea University Medical School, Swansea University, United Kingdom; 2 The Milner Centre for Evolution, Department of Biology and Biochemistry, University of Bath, United Kingdom; 3 MRC CLIMB Consortium, United Kingdom; 4 The Biostatistics Center, Kurume University, Fukuoka, Japan; 5 Brewdog PLC, Balmacassie Industrial Estate, Ellon, Aberdeenshire, United Kingdom; 6 Bioscientia Labor Ingelheim, Institut für Medizinische Diagnostik GmbH, Ingelheim, Germany; 7 Department of Zoology, University of Oxford, United Kingdom; 8 Wellcome Trust Centre for Human Genetics, Oxford, United Kingdom; 9 Institute of Farm Animal Genetics, Friedrich-Loeffler-Institut (FLI), Neustadt, Germany; 10 Department of Mathematics and Statistics, University of Helsinki, Finland; 11 Department of Biostatistics, University of Oslo, Norway; 12 The Roslin Institute and Centre for Infectious Diseases, University of Edinburgh, United Kingdom

**Keywords:** *Staphylococcus*, genomics, poultry infection, evolution, recombination

## Abstract

*Staphylococcus aureus* are globally disseminated among farmed chickens causing skeletal muscle infections, dermatitis, and septicaemia. The emergence of poultry-associated lineages has involved zoonotic transmission from humans to chickens but questions remain about the specific adaptations that promote proliferation of chicken pathogens. We characterized genetic variation in a population of genome-sequenced *S. aureus* isolates of poultry and human origin. Genealogical analysis identified a dominant poultry-associated sequence cluster within the CC5 clonal complex. Poultry and human CC5 isolates were significantly distinct from each other and more recombination events were detected in the poultry isolates. We identified 44 recombination events in 33 genes along the branch extending to the poultry-specific CC5 cluster, and 47 genes were found more often in CC5 poultry isolates compared with those from humans. Many of these gene sequences were common in chicken isolates from other clonal complexes suggesting horizontal gene transfer among poultry associated lineages. Consistent with functional predictions for putative poultry-associated genes, poultry isolates showed enhanced growth at 42 °C and greater erythrocyte lysis on chicken blood agar in comparison with human isolates. By combining phenotype information with evolutionary analyses of staphylococcal genomes, we provide evidence of adaptation, following a human-to-poultry host transition. This has important implications for the emergence and dissemination of new pathogenic clones associated with modern agriculture.

## Introduction

The expansion of the global poultry industry and rapid increase in the number of chickens presents opportunities for increased incidence of zoonotic diseases. Staphylococci are among the leading agents of bacterial infection in chickens (Peton and Le Loir 2014) and *Staphylococcus aureus* causes a wide range of chicken diseases, including septic arthritis, subdermal abscesses, and gangrenous dermatitis (Bystron et al. 2010). Molecular epidemiology studies have described highly structured *S. aureus* populations with clusters of related isolates, grouped into clonal complexes (CCs) that share five or more alleles at seven MLST loci (Enright and Spratt 1999; Urwin and Maiden 2003). Clonal complexes vary in the range of sources from which they have been isolated. For example, isolates belonging to CC385 have not been previously identified among human and mammalian species samples but have been principally found in birds including poultry (Lowder et al. 2009). Isolates from other lineages, such as CC5 and CC398, have been frequently isolated from chickens, humans, and other hosts (Abdelbary et al. 2014; Monecke et al. 2013; Price et al. 2012). CC5 is of particular concern in food production as it is the most frequent disease-causing lineage in chickens (Bystron et al. 2010; Hasman et al. 2010; Lowder et al. 2009).

Host association has previously been described in staphylococci (Shepheard et al. 2013). Global dissemination and the high level of genetic diversity observed among CC5 isolates from humans suggests long-term association with the human host (Nubel et al. 2008). The emergence of CC5 as a common lineage in poultry (Monecke et al. 2013) is thought to be the result of a single recent human-to-poultry host jump approximately 40 years ago (Lowder et al. 2009). In common with bovine-adapted *S. aureus* lineages (Herron-Olson et al. 2007), host transition of poultry CC5 isolates has been accompanied by genetic changes, including the loss of several genes involved in human disease pathogenesis and acquisition of novel mobile genetic elements from an avian-specific accessory gene pool (Lowder et al. 2009). Host-associated genetic variation of this kind is influenced by various factors. These include: (i) genetic bottlenecking leading to reduced genetic diversity in the founder population; (ii) diversification in allopatry following isolation from the ancestral population; and (iii) adaptation involving the acquisition of genomic elements that provide a competitive advantage in the new niche.

There are several examples of host adaptation following zoonotic transmission of *S. aureus* (Guinane et al. 2010; Senghore et al. 2016; Viana et al. 2010, 2015; Weinert et al. 2012), including acquisition of genes that contribute to transmission, colonization, and virulence (Wiedenbeck and Cohan 2011; Yan et al. 2016). For example, it is believed that methicillin-susceptible *S. aureus* CC398 acquired the methicillin resistance cassette in livestock before reinfecting humans (Anukool et al. 2011; Lewis et al. 2008; Price et al. 2012; Verkade et al. 2014; Ward et al. 2014; Wendlandt et al. 2014a, 2014b). Identifying host adaptations becomes particularly important when they promote the spread of disease. This is the case with plasmids that have contributed to strain virulence and conferred antibiotic resistance (pT181, pT127, pC194, pC221, pC223, and pUB112) (Ehrlich 1977) among *S. aureus* isolates causing a wide range of chicken diseases that are particularly robust and difficult to treat (Bystron et al. 2010).

Comparison of individual *S. aureus* isolates from human and chicken has been instructive in identifying host-associated genetic elements (Lowder et al. 2009). However, it remains difficult to differentiate genetic changes associated with bottlenecking and drift from those that confer an advantage in the poultry niche. Evidence for a role in adaptation is provided if specific genomic changes occur in divergent lineages that are not present in their common ancestor (homoplasy). In this study, we build on the work by Lowder et al. (2009) which identified poultry-associated plasmid and phage elements, and conduct population genomic analysis of the emergence of disease-associated *S. aureus* in chickens*.* We: (i) conduct a survey of disease causing *S. aureus* in chickens; (ii) characterize the population structure of disease-associated strains and investigate the abundance of CC5; (iii) compare chicken-disease-associated isolate genomes with clonally related bacteria from human hosts; (iv) identify the genetic elements associated with the emergence of disease; and (v) quantify homologous and accessory genome recombination in populations of *S. aureus* from different sources and genetic backgrounds. By characterizing the evolutionary events in CC5 isolates that accompanied the colonization of chickens from humans, and comparison with phylogenetically divergent *S. aureus* lineages, we provide evidence for the genetic basis of poultry adaptation. Relating adaptive hotspots to gene function, and using laboratory phenotyping assays, we describe an evolutionary history of rapid avian host adaptation of a globally disseminated animal pathogen.

## Materials and Methods

### Isolate Collection

In total, 191 isolates were sampled from diseased chickens from various breeder farms predominantly in the UK (*n* = 161), and also the USA (*n* = 25) and the Netherlands (*n* = 5) (see supplementary table S1, Supplementary Material online). Samples were collected from chickens suffering from infections of the leg (153), liver (8), footpad (7), bone marrow (4), yolk sac (4), peritoneum (3), brain (3), kidney (1), pericardium (1), spleen (1), lung (1), from a day old chick, and four samples from the surrounding environment. Samples were collected between 2008 and 2013. *S. aureus* were identified, subcultured and transported on slopes of Dorset’s egg medium before storing at −80 °C. All isolates were sequenced and 165 assembled genomes were analyzed.

### Genome Sequencing

*Staphylococcus* isolates were cultured on Columbia blood agar plates at 37 °C for 24 h. Single-colony cultures were harvested and resuspended in 3 ml of peptone yeast extract medium to minimize clumping and incubated at 37 °C with overnight shaking. DNA was extracted using the QIAamp DNA Mini Kit (QIAGEN, UK), using manufacturer’s instructions with the addition of 1.5 μg/μl lysostaphin (Ambi Products, USA) to facilitate cell lysis. The DNA of 224 isolates was sequenced at the Wellcome Trust Centre for Human Genetics (Oxford, UK) using a HiSeq 2500 (Illumina, USA). The 100 bp short read paired-end data was assembled using the *de novo* assembly algorithm, Velvet (Zerbino and Birney 2008) (version 1.2.08). The VelvetOptimiser script (version 2.2.4) was run for all odd k-mer values from 21 to 99. The minimum output contig size set to 200 bp with the scaffolding option switched off; all other program settings were left unchanged. For comparison, we also sequenced several human isolates from wound infections (*n* = 13), prosthetic joint infections (*n* = 31), nasal swabs (*n* = 9) and the SH1000 lab strain. Overall, the average number of contiguous sequences (contigs) for all 224 genomes sequenced in this study was 77 which gave rise to an average total assembled genome size of 2,893,678 bp and an average N50 of 337 Kbp. Short reads are available from the NCBI short read archive (SRA) associated with BioProject: PRJNA312437.

Our collection of avian isolates was augmented with published genomes from NCBI (*n* = 189) and further isolates were sequenced to compliment our collection. Twenty-four additional avian isolates were sequenced by the Edinburgh genomics facility at the Roslin Institute using an Illumina MiSeq (USA), including eight isolates from CC385—a clonal complex that has not previously been found outside of avian hosts (ENA project accession: PRJEB18782; average number of contigs: 382, average genome size: 2,967,259, average N50: 52 Kbp). In total, 432 *S. aureus* genomes (198 from poultry, 228 from humans, and 6 from ruminants) were used to investigate the association of genomic elements with human or poultry hosts (individual accession numbers can be found in supplementary table S1, Supplementary Material online).

### Genealogical Studies

A reference pan-genome approach (Meric et al. 2014) with gene-by-gene alignment (Maiden et al. 2013; Sheppard et al. 2012) was implemented using the open source Bacterial Isolate Genome Sequence Database: BIGSdb (Jolley and Maiden 2010), which included functionality to call MLST profiles defined by the pubMLST database (https://pubmlst.org/saureus; last accessed March 10, 2017). The publicly accessible genomes used in this study are archived in the Sheppard Lab Staphylococcal database and can be accessed at: http://www.sheppardlab.com/resources (last accessed March 10, 2017). The annotated genome of ED98 (accession NC_013450.1) and three plasmids (pAVY, pAXY, and pT181) were combined and used as a reference genome (Meric et al. 2014). The BLAST algorithm was used to scan all genomes for gene orthologs at each locus in the reference genome. An ortholog was defined as a reciprocal best hit of the sequence with >70% nucleotide identity over 50% of the alignment length. MAFFT software was used to align gene orthologs on a gene-by-gene basis, and these data concatenated into contiguous sequence for each isolate genome, including gaps (Sheppard et al. 2013). Alignment based on the ED98 reference genome and poultry plasmids was constructed (2,780 genes) and a heuristic maximum-likelihood tree generated using FastTree2 (version 2.1.0) (Price et al. 2010) with the generalized time reversible substitution model enabling reconstruction of branch lengths >0.0000005 (1,000 times higher than the default FastTree parameters). In addition, gene-by-gene alignments were extracted from the BIGSdb for individual genes present in CC5 poultry isolates and completely absent from CC5 human isolates. The RAST automated annotation server was used to predict putative gene function (Aziz et al. 2008).

### Core Genome Recombination

Isolate genomes (*n* = 432) were aligned using MAFFT (Katoh et al. 2002). Mutations found in single isolates were excluded, as they carry no information about the shared ancestry between lineages and a total of 279,646 SNPs were recorded in the isolates. Isolates were grouped into clusters through hierBAPS analysis (version 6.0)(Cheng et al. 2013) of the resulting core genome alignment using five replicate runs with the upper bound for the number of clusters varying between 50 and 100. All estimation runs converged to the same estimate of the posterior mode clustering, indicating strongly peaked posterior distribution. CC5 was identified as one of the hierBAPS clusters and subsequently, BratNextGen (BNG) software (Marttinen et al. 2012) was used to estimate the amount of homologous recombination in the core genome for the 68 isolates assigned to that cluster. Within BNG, 20 iterations of hidden Markov model parameter estimation were performed and 30 groups of isolates were identified. Statistically significant (*P* value < 0.05) recombination in the core genome was determined with 100 parallel permutation runs. We report the ratio at which changes in nucleotide sequence are introduced by recombination relative to mutation (r/m) as in McNally et al. (2013).

### Inference of Recombination Regions

ClonalFrame infers the clonal relationship of bacteria and the chromosomal position of homologous recombination events that disrupt a clonal pattern of inheritance (Didelot and Falush 2007). Gene-by-gene alignment (XMFA) files of all ED98 and plasmid genes present in each of the *S. aureus* strains were generated. A genealogy for these alignments was estimated using ClonalFrame (version 1.2) on concatenated sequences of 46 CC5 genomes with 100,000 iterations, half of which were discarded as burn-in. Substitution mutation and recombination regions were categorized from the output of ClonalFrame. The posterior probability of recombination and substitution at each site is calculated by ClonalFrame and recombination events were defined with a probability of recombination more than 75%, reaching 95% at any one site.

### Phenotype Assays

#### Growth at Human and Avian Body Temperature

Twelve *S. aureus* CC5 poultry isolates were selected for comparison with four representative *S. aureus* human isolates (from clades containing poultry isolates: two CC5 and two CC1) to assess their growth under human (37°C) and avian body temperatures (42°C). Isolates were cultured on CBA plates at 37°C overnight prior to inoculation of 3 ml Mueller-Hinton broth with shaking at 37°C overnight until stationary phase (OD_600_ between 1.0 and 1.5). Aliquots were diluted 1:100 in fresh Mueller-Hinton medium and 195 µl of each sample loaded into a 96-well plate with absorbance read every hour using the FLUOstar Omega microplate reader (BMG Labtech, Germany) at 600 nm. Experiments were performed in triplicate and the average of three readings for each run was calculated. We performed an extra sum-of-squares *F*-test (*P* value threshold of 0.05) to test the null hypothesis that a single sigmoid regression curve fits the distribution of both human and poultry samples at human and avian body temperature.

#### Erythrocyte Lysis Assays

The same isolates used in growth experiments were also used to assay for their ability to digest chicken and human erythrocytes. Isolates were cultured on CBA plates at 37°C overnight prior to inoculation of 3 ml Mueller-Hinton broth with shaking at 37°C for up to 3 h until late exponential phase (OD600 ∼0.6). Each sample was diluted 1:10 using fresh Mueller-Hinton broth and 2 µl added to wells of a six-well plate containing 3 ml of defibrinated chicken blood (Rockland antibiotics and assays, USA #R102-0100) or pooled whole human blood (collected using haematology tubes with EDTA from volunteer scheme at Swansea University). Plates were incubated at 37°C for 48 h and positive results recorded for erythrocyte lysis if a halo (>1 mm) was measured around the colony. Unpaired *t*-tests were used to evaluate the significance of differences between human and chicken isolates, in addition to isolates with and without the Staphostatin B gene.

**Table 2:** Lysis of Chicken and Human Erythrocytes on Columbia Blood Agar (Cheung et al. 2012) Plates with *S. aureus* Isolates from Humans (*n* = 4) and Poultry (*n* = 12)

Isolate ID	Alias	Host Species	Clonal Complex	Staphostatin B (SAAV_C21) Gene Presence	Lysis on Human Blood Agar	Lysis on Chicken Blood Agar
4	N315	Human	5	x	✓	x
343	SS_0499	Human	5	x	X	x
437	SS_0017	Human	1	x	✓	x
529	SS_0119	Human	1	x	✓	✓
349	SS_0542	Poultry	5	✓	x	✓
351	SS_0544	Poultry	5	✓	✓	✓
385	SS_0578	Poultry	5	✓	x	✓
386	SS_0579	Poultry	5	✓	x	✓
388	SS_0581	Poultry	5	x	x	✓
815	SS_0593	Poultry	5	x	✓	✓
845	SS_0623	Poultry	5	x	✓	✓
853	SS_0631	Poultry	5	✓	✓	✓
875	SS_0653	Poultry	5	x	x	x
907	SS_0684	Poultry	5	✓	✓	✓
911	SS_0688	Poultry	5	✓	✓	✓
930	SS_0707	Poultry	5	✓	✓	✓

Note.—A positive result was recorded if a halo of lysis was observed around the colony after 24 h, experiments were performed in triplicate.

## Results

### Emergence and Rapid Clonal Expansion of CC5 in Chicken

**Fig. 1.—:**
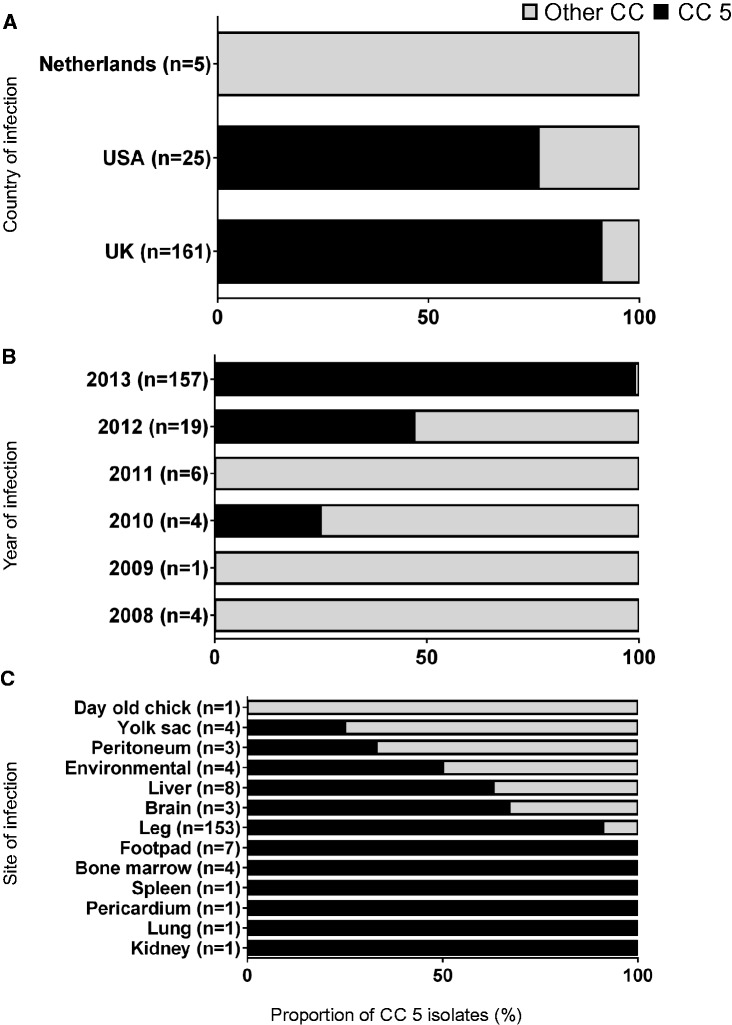
Samples were collected from infected breeder chickens across the UK (*n* = 161), USA (*n* = 25), and the Netherlands (*n* = 5). Grouped bar charts show the relative proportion of isolates belonging to CC5 by country (1*a*), year of collection (1*b*), and disease site (1*c*). MLST clonal complexes were assigned based on shared sequence at five or more MLST house-keeping loci.

The majority of avian isolates collected belonged to CC5 (85%). Isolates collected from the UK (91%) and the USA (76%) were predominantly of CC5, but no CC5 isolates were collected from the Netherlands, although these five isolates represent only a small proportion of our collection (fig. 1*a*). Isolates in this study are predominantly from 2012 to 2013 and are dominated by isolates from CC5 (fig. 1*b*). Samples from all disease types studied contained CC5 isolates (fig. 1*c*) and the only other CC detected in our collection was the livestock-associated CC398. As in other poultry *S. aureus* studies (Lowder et al. 2009), CC5 is the dominant lineage within our collection. Further long-term surveillance is necessary to establish the timescale of emergence of this lineage.

**Fig. 2.—:**
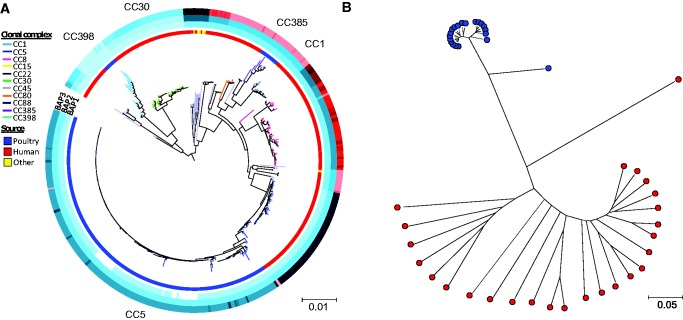
Genetic relatedness of *S. aureus isolates* from different hosts. (*a*) Host origin of all *S. aureus* isolates from chicken (blue), human (red), and other species (yellow). Clonal complex (CC) designations are based on shared MLST housekeeping loci. Chicken isolates were found in five sequence clusters, corresponding to CCs 1, 5, 30, 358, and 398 which are highlighted. The tree was constructed from a core genome alignment (2,789,909 bp) of 1,700 genes using an approximation of the maximum likelihood algorithm. (*b*) Reconstruction of the clonal frame with putative recombination regions removed of CC5, including 20 chicken (blue) and 26 human (red) isolates (2,302,773 bp alignment in ClonalFrame).

A maximum-likelihood phylogenetic tree of all 432 isolates was constructed using a reference pan-genome combining the ED98 isolate genome and three plasmids previously identified from poultry: pAVX (17,256 bp); pAVY (1,442 bp); and pT181 (4,439 bp) (Lowder et al. 2009; Meric et al. 2014) (fig. 2*A*). Four distinct lineages contained isolates from poultry (Price et al. 2010) and within the CC5 sequence cluster a clear poultry sublineage was evident (there was also a single poultry isolate belonging to the CC30). Hierarchical Bayesian estimations of the genetic population structure (hierBAPS) also grouped poultry and human isolates within the CC5 cluster.

### Increased Recombination in CC5 Poultry Isolate Core Genomes

The number of ancestral populations was estimated by grouping isolates into genetically divergent clusters using BratNextGen (Marttinen et al. 2012). The algorithm inferred the positions and sizes of DNA sequence segments with evidence of homologous recombination and grouped isolates according to recombination pools (see supplementary fig S1, Supplementary Material online). At the highest level of BAPS clustering there were 30 *S. aureus* sequence clusters composed of 2 or more isolates (fig. 2*a*). Recombination was unevenly detected across *S. aureus* sequence clusters (see supplementary fig S1, Supplementary Material online). The rate at which recombination introduced nucleotide changes, relative to mutation (r/m), in the CC5 complex was 1.08. Disproportionally more recombination events were identified in the chicken CC5 isolates ($\overset{-}{x}$ = 34.6 per isolate) compared with the human CC5 isolates ($\overset{-}{x}$ = 18.5). In order to assess the statistical significance of this we assumed independence of the isolates and estimated the probability of finding at least one recombination event in an isolate from each population. In human isolates, there were 40/59 isolates with at least one recombination event and in poultry there were 9/9. The z-score test statistic was 2.005 and a *P* value for a one-sided test was 0.022, which was significant at 5% level.

### Poultry-Associated Core-Genome Recombination

A subset of CC5 isolates (human and poultry) were selected for further analysis with ClonalFrame (Didelot & Falush 2007), which infers the clonal relationship of bacteria and the chromosomal position of homologous recombination events. Inferred regions of recombination were removed (and exported) prior to phylogenetic reconstruction of the clonal frame (fig. 2*b*). Execution of ClonalFrame with 46 *S. aureus* strains (20 CC5 poultry and 26 CC5 human) gave rise to a genealogy that also demonstrated complete separation of the poultry and human isolates within the CC5 complex. In comparison to the isolate phylogeny prior to the removal of recombination, there is tighter clustering of the poultry CC5 isolates, which supports our observation that CC5 poultry isolates have been subject to more recombination.

ClonalFrame inferred 196 substitution sites and 44 recombination regions mapping to 33 genes (see supplementary table S2, Supplementary Material online). BLAST was used to locate exact matches (100% identity) of the recombination regions (between 6 and 913 bp in length) in poultry isolates from other clonal complexes (see supplementary table S2, Supplementary Material online). The shortest recombination region detected by ClonalFrame was 6 bp in length located within *SAAV_2007* (*hlb*, β-haemolysin) and upstream of the phage-associated virulence factor *SAAV_2008* (ornithine cyclodeaminase) (Lowder et al. 2009; Price et al. 2012) and was similar to a ribosome-binding site (TTATAA). This recombination region was too short to be detected using a standard BLAST search, and was manually identified in all CC1, CC5, CC385, and CC398 poultry isolates, but was completely absent from human isolates.

Quantification of the poultry associated recombination regions in poultry and human isolates was carried out for each clonal complex (see supplementary table S2, Supplementary Material online). In all four lineages studied here (CC1, CC5, CC385, and CC398), the putative recombination regions were detected more often in isolates obtained from chicken compared with those from humans. All recombination regions (43/44 by BLAST plus the short 6 bp recombination region) were found in CC5 poultry isolates (*n* = 177), but only 31 of those were present in the human CC5 isolates (*n* = 53) (see supplementary table S2, Supplementary Material online). In CC1, 35 recombination regions were found in chicken isolates (*n* = 3) compared with 24 in the isolates from humans (*n* = 11). Many of the inferred recombination regions were found in isolates from both chicken and human sources in the livestock-associated CC398 clonal complex, 38 in chicken isolates (*n* = 9) and 28 in the human CC398 isolates (*n* = 8). Forty-three recombination regions were found in chicken isolates (*n* = 8) from the poultry ancestral clonal complex CC385 (no human isolates). In total, 31 recombination regions were found more often in chicken isolates in all four of the investigated clonal complexes, consistent with ongoing poultry adaptation. A summary of the relative abundance of all poultry-associated genes and recombination regions found by analysis of CC5 is presented in Table 1.

**Table 1:** Prevalence of Chicken-Associated Genes and Recombination Regions as Defined by Clonal Frame Analysis of CC5 Isolates

Poultry-Associated Genetic Variation^a^	Prevalence (%)							
	CC5		CC398		CC1		CC385	
	Poultry (*n* = 177)	Human (*n* = 50)	Poultry (*n* = 9)	Human (*n* = 9)	Poultry (*n* = 3)	Human (*n* = 18)	Poultry (*n* = 8)	Human (*n* = 0)
Genes	98	0	65	4	55	2	62	—
Recombination regions	90	15	49	45	70	12	62	—

a Genes and recombination regions found to be associated with CC5 poultry isolates.

### Poultry Associated Accessory Genes

Comparison of gene content revealed that 2,716 genes from the poultry-associated reference pan-genome were shared between CC5 human and CC5 poultry isolates. No genes were found exclusively in CC5 human isolates and 47 genes were found predominantly (≥95%) in CC5 poultry but absent in human isolates (see supplementary table S2, Supplementary Material online). Thirty-eight of the 47 genes were also present in CC1 poultry isolates with variable frequency from 33% to 100% (see supplementary table S2, Supplementary Material online). Forty-one genes were present in 22–89% of CC398 poultry isolates (see supplementary table S2, Supplementary Material online). All 47 poultry-associated genes were present in CC385 poultry isolates to some degree (13–100%) (see supplementary table S2, Supplementary Material online). Only one poultry-associated gene, *SAAV_0809*, which belongs to the *S. aureus* pathogenicity island (SaPI), was present in 82% of CC1 human isolates. Thirteen genes were found in 13–25% of CC398 human isolates (see supplementary table S2, Supplementary Material online). In total, 36 of the genes associated with poultry in CC5 were present more often in poultry isolates from all four investigated clonal complexes.

### Colocalization of Poultry-Associated Genes and Recombination Regions in Three Recombination Hotspots

**Fig. 3.—:**
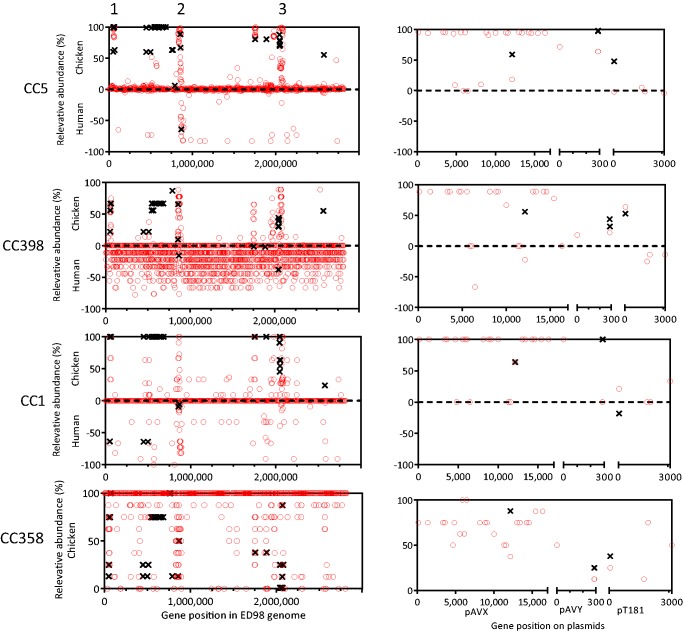
Genes and recombination regions identified as poultry-associated in ClonalFrame analysis mapped to the ED98 reference genome and three plasmids (pAVX, pAVY, and pT181). The frequency of these genes (red circles) and recombination regions (black crosses) in chicken and human isolate genomes is shown for CC5, CC398, CC1, and CC385 (chicken only). The relative abundance of these genes/recombination regions was calculated as presence in chicken minus presence in human isolates. 0 score denotes equal presence in poultry and human isolates. The majority of poultry-association in the core and accessory genome is colocalized in three chromosomal regions, which are labeled.

Thirty-three genes containing homologous recombination regions and 47 poultry-associated accessory genes were mapped to the ED98 reference genome and to three plasmids (pAVX, pAVY, and pT181). Where sequence mapped to regions of unknown function, for example genes encoding hypothetical proteins, the locus ID from ED98 was recorded and the putative gene function was investigated using RAST and BLAST comparison with the NCBI database (see supplementary table S2, Supplementary Material online). Three genes (*SAAV_A2, SAAV_B1*, and *SAAV_C17*) containing recombination regions were mapped to locations on the three poultry-associated plasmids, pAVY, pT181, and pAVX (Lowder et al. 2009). Nine poultry associated genes were mapped to pAVX (*SAAV_C01, SAAV_C03, SAAV_C04, aurT, scpB, SAAV_C12, SAAV_C15, SAAV_C18*, and the *pemK*-like *SAAV_20*). Many of the 80 genes located on the reference genome clustered into three distinct genomic regions (fig. 3), including genes with putative roles in heat shock response, haemolysis, adhesion, mobile elements, and transposons (see supplementary table S2, Supplementary Material online).

**Fig. 4.—:**
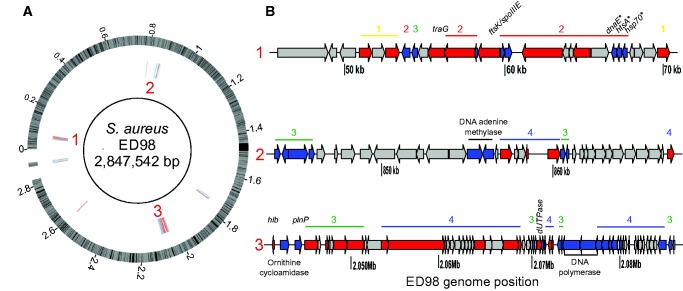
Genome positions of colocalized poultry-associated genes and recombination regions. (*a*) Poultry-associated genes (blue) and recombination regions (red) mapped to the ED98 reference chromosome. Hot spots of colocalization of poultry-associated elements are numbered. (*b*) Schematic diagrams of each hot spot showing gene content, including poultry-associated genes and genes containing recombination regions. Genes with no poultry-association in the same regions are also shown (grey). Poultry-associated genes and genes containing recombination regions are labeled, including *S. aureus* pathogenicity island genes (1), transposon-related genes (2), hypothetical proteins (3), and phage-related genes (4). More details of putative gene function can be found in supplementary table S2, Supplementary Material online.

A total of 58 poultry-associated genes and genetic elements are predicted to be involved in the transfer of mobile genetic elements, phage proteins, and hypothetical proteins (fig. 4*a*, see supplementary table S2, Supplementary Material online). Putative gene function was inferred using the RAST annotation server (Aziz et al. 2008) and BLAST sequence comparisons made against the NCBI database. The genes included in region 1 map to a similar location on the ED98 reference genomes and may form a transposon comprised of 16 genes with predicted function including hypothetical proteins, lipoproteins a transposase, a plasmid-related conjugal transfer protein (TraG, SAAV_0051) and an FtsK/SpoIIIE family protein (SAAV_0054) (fig. 4*b*). Regions 2 and 3 are phage-related. Genes encoding hypothetical proteins, phage proteins, the fibronectin-binding protein (Fnb, SAAV_2566) and a putative β-haemolysin (SAAV_2007) were found in region 3. The β-toxin gene is common among *S. aureus* isolates, but often truncated by insertion of a beta-converting phage in most human infection isolates (Salgado-Pabon et al. 2014). The three poultry-associated plasmids were composed of several hypothetical proteins, a replication associated protein (SAAV_C12), the staphylococcal virulence factor Staphostatin A (ScpB, SAAV_C09), and the plasmid-retention PemK-like protein (SAAV_C20).

### Poultry Isolates Show Enhanced Growth and Erythrocyte Lysis at Avian Body Temperature on Chicken Blood Agar

**Fig. 5.—:**
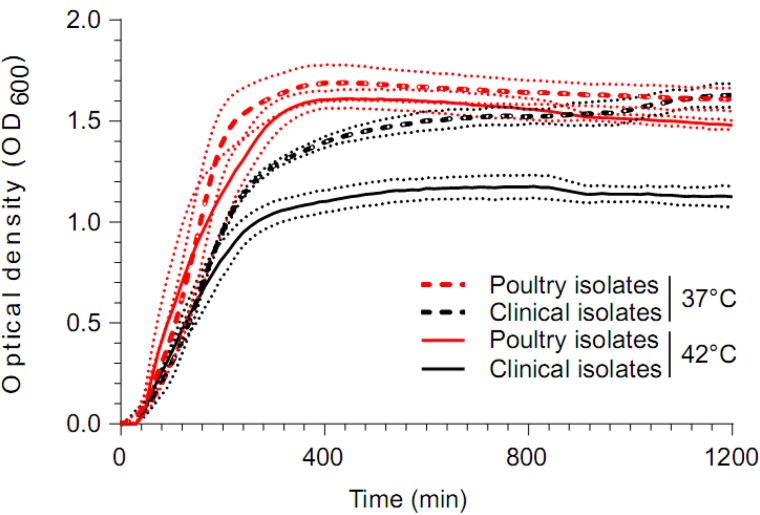
Growth of *S. aureus* isolates from poultry (red) and human (black) in TSB medium. Curves represent growth levels in vitro (OD_600_) over a period of 20 h at 37 °C (dashed lines) and 42 °C (solid lines) in medium. Mean growth levels and standard deviation (dotted lines) was calculated for 12 poultry and four human clinical samples.

A subset of CC5 poultry (12) and human (4) isolates were grown at mammalian (37 °C) and poultry body temperatures (42 °C) overnight. The poultry isolates reached a peak OD_600_ of 1.8, whereas the human isolates grew to a similar OD_600_ of 1.7 when grown at 37 °C. However, when grown at 42 °C the poultry isolates grew to a similar density (OD_600_ of 1.8) but the human isolates did not (OD_600_ of 1.4; fig. 5). Using an extra sum-of-squares *F*-test (*P* value threshold of 0.05) the distribution of human isolates at both temperatures could be fitted to a single sigmoid regression curve. This null hypothesis was rejected when comparing poultry isolates, which showed significant difference when grown at avian and human body temperatures.

The ability to lyse chicken erythrocytes was tested on a subset of isolates. Most of the isolates from chicken (11/12) lysed chicken erythrocytes compared with only one (out of four) of the isolates from humans. Conversely, most of the human isolates (3/4), but only around half (7/12) of the poultry isolates were able to lyse erythrocytes on blood agar plates with human blood (Table 2). The differences observed between human and poultry isolates on chicken blood agar were statistically significant (*P* value = 0.0048, unpaired *t*-test), but the results using human blood agar were not (*P* value > 0.05). Several poultry-associated genes have been implicated in increased pathogenicity in chicken (Abdalrahman et al. 2015; Lowder and Fitzgerald 2010), including *scpB* (Staphostatin A) which encodes a putative cysteine protease (Lowder et al. 2009; Takeuchi et al. 1999) and is located on the pAVX plasmid (Takeuchi et al. 2002) previously identified in studies of poultry infection. All eight isolates containing this gene lysed chicken blood in Columbia Blood Agar (Cheung et al. 2012). Half of the isolates (1/4 human and 3/4 poultry) not carrying the gene lysed chicken blood (Table 1). Only the differences observed on chicken blood agar were statistically significant (*P* value = 0.0192, unpaired *t*-test).

### The Poultry Ancestral Lineage CC385 Exhibits More Genetic Diversity than CC5

All of the 47 poultry-associated genes and alleles identified in CC5 were also found in at least one other clonal complex. Individual gene phylogenies were constructed for all 47 genes from all 197 poultry isolates, including isolates from CC1, CC5, CC385, and CC398 (see supplementary fig S2, Supplementary Material online). The average branch length per isolate was estimated for each clonal complex (see supplementary table S3, Supplementary Material online). CC385 is considered an avian-associated lineage, only isolated from bird species. Longer association with the host results in increased genetic diversity through mutation and genetic drift. We therefore tested if average branch length is shorter in CC5 isolates, which are believed to have colonized more recently. In total, the number of polymorphic sites per isolate was much lower for CC5 (867) than for isolates from CC385 (2012) and CC398 (2505). The total number of unique/shared alleles for the 47 poultry-associated genes was similar across all clonal complexes where there was more than one isolate in this study (see supplementary table S3, Supplementary Material online).

## Discussion

Genealogical analysis of the isolates in CC5 identified a sublineage of poultry-associated isolates. This is consistent with a scenario where chicken colonization by CC5 resulted from a host transition event from a human ancestral population (Lowder et al. 2009). The majority of poultry disease isolates in this study belonged to CC5 and the widespread distribution of this clonal complex among poultry is consistent with the emergence of an important disease-causing lineage following the human-to-poultry host jump (fig. 1) (Bystron et al. 2010; Hasman et al. 2010; Lowder et al. 2009).

Reconstruction of the clonal frame following removal of inferred recombination regions, further supports this hypothesis demonstrating complete segregation of the poultry and human CC5 isolates in this study and the emergence of a single lineage from an ancestral genetically diverse human CC5 population (fig. 2). This pattern of genetic bottlenecking and the emergence of epidemic clones from a founder population are well established in *S. aureus*, for example in the spread of globally disseminated healthcare-associated methicillin-resistant *S. aureus* (MRSA) clones (Hsu et al. 2015). Although the impact of clinical practice and antibiotic usage have been well studied in relation to the evolution of nosocomial pathogen populations (Enright et al. 2000; Gill et al. 2005; Hiramatsu et al. 2001; Mwangi et al. 2007; Wertheim et al. 2005), less is known about the emergence of disease-associated staphylococci in agriculture. Rapid dissemination of clones is likely facilitated by global food production networks, but to identify the specific genomic changes related to the emergence of pathogenic lineages, it is instructive to use a comparative functional genomics approach.

Adaptation is mediated by recombination in *S. aureus* and our estimate of the rate at which recombination is introducing nucleotide changes, relative to mutation in CC5 (r/m = 1.08), is consistent with previous estimates based on core genomes in ST-239 (r/m = 1.13 Castillo-Ramirez et al. 2012) and several lineages (STs 38, 36, 22, and 12) by MLST (r/m = 1.1 by Feil et al. 2003). Different recombination rates have been observed between *S. aureus* lineages with admixture occurring between some, but not all clonal complexes (Castillo-Ramirez et al. 2012; Meric et al. 2015). There is also variation in recombination estimates within CC5 human isolates (Duchêne et al. 2016; Everitt et al. 2014; Meric et al. 2015). In this study, the amount of realized recombination in CC5 poultry isolates is almost double that inferred in isolates from humans. This is consistent with a scenario of adaptation mediated by recombination following colonization of a new niche.

Characterization of the genetic changes associated with the divergence of CC5 poultry isolates from human CC5 isolates identified genomic variations that may have been involved in adaptation to poultry. Broadly, these can be considered as differences in the core and accessory genome compared with CC5 isolates from humans. Whole genome comparison of CC5 isolates identified 47 genes found in >95% of poultry isolates but absent from human isolate genomes (see supplementary table S2, Supplementary Material online). The predicted function of these genes included staphylococcal virulence factors, such as toxin production, adhesion, stress response, plasmid maintenance and antibiotic resistance, as well as mobile genetic elements. For accessory genome characterization, it was necessary to compare CC5 genomes with the chicken *S. aureus* reference strain ED98. Although this is less well annotated than some human reference strains, comparison allowed genes with orthologues of known function to be associated with the CC5 poultry accessory genome. These included: the *hlb* toxin gene which contributes to tissue damage and can influence disease severity (Bramley et al. 1989; Guinane et al. 2008); the gene encoding fibronectin-binding protein *fnbB*, which facilitates colonization and attachment (Foster and Hook 1998; Peacock et al. 2002), and mobile genetic elements involved in DNA transfer (Everitt et al. 2014; Malachowa and DeLeo 2010). Several other accessory genes (*SAAV_0806, SAAV_0807, SAAV_0808,* and *SAAV_0809*) have been identified as components of *S. aureus* pathogenicity islands (SaPI) (Viana et al. 2010). In addition to variation in accessory genes, recombination in homologous sequence can be associated with host adaptation (Didelot and Falush 2007; Sheppard et al. 2013). Inferred recombination regions (*n* = 44) in the poultry-associated CC5 isolates were mapped to 33 genes in the reference pan-genome. A total of 9% (3/33) mapped to plasmid genes, with the remaining recombinant sequences mapping to 30 chromosomal genes. The function of these genes varied including those associated with hypothetical proteins, transposition and DNA regulation.

The absence, or relative scarcity, of the 80 poultry-associated accessory genes (*n* = 47) and genes containing poultry-associated recombination regions (*n* = 33) in the ancestral human CC5 population suggests that acquisition of these elements was associated with chicken colonization. However, this alone is not confirmation of adaptation. Evidence for significance of these genes in poultry adaptation is supported by their differential presence in poultry and human isolates from other clonal complexes. The majority of CC5 poultry-associated genes/alleles were more commonly present in poultry isolates from CC398 and CC1 with only 15% being more common among isolates from humans (see supplementary table S2, Supplementary Material online). All of the CC5 poultry-associated accessory genes, and 96% of associated homologous recombination segments, were present in the analyzed CC385 isolates that have only been isolated from birds. This is consistent with an evolutionary scenario where horizontal gene transfer (HGT) occurred, introducing genetic material that has evolved in a different genetic background to *S. aureus* strains colonizing chickens. CC385 has not been previously associated with humans or mammals but has been isolated from various wild and reared birds suggesting that the CC385 lineage has had long-term avian host restriction (Lowder et al. 2009). Consistent with this, most of the genetic variation in poultry-associated genes and alleles, identified in CC5, is present within isolate genomes from other complexes (Table 2). Furthermore, in these genes there were more than twice as many polymorphisms per isolate in CC385 compared with CC5 (see supplementary and table S3, Supplementary Material online). It is possible that each CC could have acquired the genes from a human-associated lineage and then transferred to each CC at different times. However, the absence of CC structuring on individual genes phylogenies (see supplementary fig S2, Supplementary Material online), where avian alleles cluster together, implies that poultry isolates from different CCs have acquired the genes necessary for adaption to the poultry niche from preexisting avian-associated lineages.

Some evidence of the functional significance of poultry-associated genes was investigated in laboratory phenotyping assays. Three genes located in the three poultry-associated CC5 hotspots had putative functions related to chicken colonization (beta-haemolysin, *SAAV_2007*, and *SAAV_C21*). Among the most obvious environmental challenges for *S. aureus* colonizing chickens is the higher host body temperature of 42 °C in chickens compared with 37 °C in mammals. Two poultry-associated genes in CC5 isolates (*SAAV_0062* and *SAAV_0064*) had >85% nucleotide identity to genes involved in temperature-dependent growth, including *dnaK* and *dnaE* which have been shown to be important for growth at 42 °C in *S. aureus* poultry strains (Inoue et al. 2001; Singh et al. 2007). In laboratory assays, chicken CC5 isolates containing these thermo-tolerance genes demonstrated enhanced growth at avian body temperature compared with growth at 37 °C (fig. 5).

A number of the chicken associated genes potentially had a role in pathogenicity including beta-haemolysin, *SAAV_2007* and *SAAV_C21* putatively involved in haemolysis contributing to *S. aureus* pathogenicity (Kuroda et al. 2007). In laboratory assays, CC5 isolates of human origin showed very little haemolytic activity when grown on agar plates containing chicken blood. However, 11 of 12 poultry strains lysed chicken blood in agar under the same conditions (Table 1). The pAVX plasmid has previously been implicated in lysis of avian erythrocytes and contains a putative thiol protease, *scpA* (*SAAV_C10*), which—when expressed—contributes to *S. aureus* virulence (Bonar et al. 2016). Other protease genes are also likely to contribute to lysis of erythrocytes as *S. aureus* has several orthologous haemolytic genes (Jusko et al. 2014).

Sustainable food production in intensive agricultural systems is threatened by the spread of zoonotic pathogens. The recent host transition of CC5 *S. aureus* from humans to poultry has resulted in the emergence of a major pathogen that causes various diseases in chickens in agricultural systems. Comparative genomics of commensal and pathogenic staphylococci offers considerable opportunities to improve understanding of the epidemiology and evolution of these organisms (Yahara et al. 2016). Here, by identifying the evolutionary events associated with chicken colonization, we provide evidence for the role of lateral gene transfer and homologous recombination in the emergence of CC5 as a major poultry pathogen. Many of the genes involved have putative functions that could be related to adaptation to chicken, but all were present in other *S. aureus* clonal complexes isolated from chickens. This is consistent with adaptation though HGT within the resident poultry staphylococcal community, potentially leading to convergent evolution. The importance of HGT in pathogen emergence is well documented in staphylococci, for example in the emergence of MRSA in hospitals. Although it may be difficult or impossible to prevent adaptation through HGT in recombining bacteria, characterizing the genes associated with adaptation can provide important information about the genetic basis of pathogen emergence. A clearer understanding of this may provide opportunities for improving agricultural practices and targeting interventions to reduce animal disease.

**Data deposition:** Accession numbers can be found in supplementary table S1, Supplementary Material online, for all genomes used in this study. Short reads of all isolates sequenced have been deposited in the short read archive under BioProject accession PRJNA312437 (http://www.ncbi.nlm.nih.gov/bioproject/PRJNA312437). Additional genomes sequenced at the Edinburgh Genomics facility at the Roslin Institute, Edinburgh have been deposited on the European Nucleotide Archive under project accession PRJEB18782. Assembled genomes can also be accessed via the resource section of our group website (https://sheppardlab.com/resources/; last accessed March 10, 2017) in the public Sheppard Staphylococcal BIGS database server. 

Supplementary figures and tables have been deposited on figshare: https://figshare.com/articles/Recombination-med iated_host-adaptation_by_avian_Staphylococcus_aureus/3863736 (last accessed March 10, 2017).

## Supplementary Material

Supplementary data are available at *Genome Biology and Evolution* online.

Supplementary Data
